# Isolation and characterization of peritoneal microvascular pericytes

**DOI:** 10.1002/2211-5463.13386

**Published:** 2022-03-15

**Authors:** Lei Tang, Jun Shi, Manshu Yu, Yun Shan, Juan Zhao, Meixiao Sheng

**Affiliations:** ^1^ First Clinic Medical School Nanjing University of Chinese Medicine China; ^2^ 375808 Renal Division Affiliated Hospital of Nanjing University of Chinese Medicine Nanjing China; ^3^ 66478 School of Traditional Chinese Medicine & School of Integrated Chinese and Western Medicine Nanjing University of Chinese Medicine China; ^4^ 66478 Key Laboratory for Metabolic Diseases in Chinese Medicine Nanjing University of Chinese Medicine China

**Keywords:** isolation and characterization, pericyte–myofibroblast transition, pericytes, peritoneal fibrosis

## Abstract

As a potential source of myofibroblasts, pericytes may play a role in human peritoneal fibrosis. The culture of primary vascular pericytes in animals has previously been reported, most of which are derived from cerebral and retinal microvasculature. Here, in the field of peritoneal dialysis, we describe a method to isolate and characterize mouse peritoneal microvascular pericytes. The mesenteric tissues of five mice were collected and digested by type II collagenase and type I DNase. After cell attachment, the culture fluid was replaced with pericyte‐conditioned medium. Pericytes with high purity (99.0%) could be isolated by enzymatic disaggregation combined with conditional culture and magnetic activated cell sorting. The primary cells were triangular or polygonal with protrusions, and confluent cell culture could be established in 3 days. The primary pericytes were positive for platelet‐derived growth factor receptor‐β, α‐smooth muscle actin, neuron‐glial antigen 2, and CD13. Moreover, they promoted formation of endothelial tubes, and pericyte–myofibroblast transition occurred after treatment with transforming growth factor‐β1. In summary, we describe here a reproducible isolation protocol for primary peritoneal pericytes, which may be a powerful tool for *in vitro* peritoneal fibrosis studies.

## Graphical abstract

Here, we describe an isolation method for mouse peritoneal microvascular pericytes, which may be a powerful tool for the study of peritoneal fibrosis *in vitro*. Primary pericytes with high purity (99.0%) were isolated by enzymatic disaggregation followed by conditional culture and magnetic‐activated cell sorting. These pericytes have potential for promotion of endothelial tube formation, as well pericyte–myofibroblast transition.

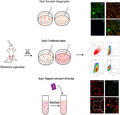

**Abbreviations**
ECM: extracellular matrix
HRMEC: human retinal microvascular endothelial cell
HRMPC: human retinal microvascular pericyte
MACS: magnetic activated cell sorting
MPVEC: mouse peritoneal vascular endothelial cell
MPVPC: mouse peritoneal vascular pericyte
MyoFs: myofibroblasts
NG2: neuron‐glial antigen 2
PDGFR‐β: platelet‐derived growth factor receptor‐β
TGF‐β1: transforming growth factor‐β1
α‐SMA: α‐smooth muscle actin

Peritoneal dialysis is the main therapy for patients with end‐stage renal disease, which relies on ultrafiltration of the peritoneum. With the progression of dialysis, however, peritoneal structure remodeling occurs as a result of exposure to conventional peritoneal dialysis solutions. Various pathological factors lead to aggravating peritoneal fibrosis, decreasing dialysis efficiency, and eventually ultrafiltration failure. The activation and proliferation of myofibroblasts (MyoFs), which are the main component of the mesothelial matrix [1], play a central role in the pathological process of peritoneal fibrosis.

In the environment of glucose degradation products [2], uremic toxins [3], and inflammation [4], MyoFs proliferate to synthesize and secrete extracellular matrix (ECM) and overexpress α‐smooth muscle actin (α‐SMA), which mediates collagen deposition and fibronectin formation. During this process, ECM levels increase significantly, although their origin is still controversial. Researchers have focused on interstitial fibroblasts [5], a well‐studied cell type that synthesizes components of the ECM and maintains the internal environment under physiological conditions. Moreover, epithelial–mesenchymal transition has been a hot research topic in the field of peritoneal fibrosis and even fibrotic diseases since 2003, when scientists first revealed the mesenchymal characteristics of mesothelial cells in peritoneal dialysis fluid [6]. Endothelial cells are similar to epithelial cells and transform into a mesenchymal phenotype, known as the endothelial–mesenchymal transition [7]. However, with the development of cell tracking technology and single‐cell RNA sequencing, these theories have been greatly challenged. A recent study mapped, the cellular transcriptome of healthy and fibrotic human kidneys by single‐cell RNA sequencing, revealing a distinct subpopulation of pericytes as potent cellular sources of MyoFs [8].

Pericytes are mural cells located on the side of the basement membrane, and they maintain vascular stability by interacting with endothelial cells [9]. As the progenitor of interstitial myofibroblasts, activated pericytes separate from the microvasculature and transdifferentiate into MyoFs [10]. On the other hand, separation of pericytes results in elevated microvascular permeability and structural degeneration, which is a potential pathology of fibrosis [11]. Thus, pericyte–myofibroblast transition may be a new approach regulating MyoFs in peritoneal fibrosis. At present, there are no stable peritoneal cell lines or mature extraction technologies of pericytes, which has brought obstacles and challenges to the research of peritoneal fibrosis. In the present study, we have developed an efficient protocol for the isolation and characterization of mouse peritoneal microvascular pericytes.

## Materials and methods

### Preparation of murine mesentery

Wild‐type C57BL/6 mice (Vital River Technology, Beijing, China) at 6 weeks of age (random sex selection) were maintained under a 12 : 12 h light/dark photocycle at a relatively constant room temperature (22 °C on average) and given free access to food and water during the acclimation periods. Then, mice were killed and sterilized and each animal was laid supine on ice and preoperative hair removal was performed. To fully expose the mesentery, a cross incision was performed from the underbelly up to the sternum. Then, the total mesenteric tissue including the vascular bed, was separated from the bowel and stored in ice‐cold phosphate‐buffered saline (Invitrogen, Carlsbad, CA, USA) with 1% penicillin–streptomycin. Animal experiments and procedures were performed according to the Guidelines for the Care and Use of Laboratory Animals (1985, NIH) after review by the Ethics Committee in the Affiliated Hospital of Nanjing University of Chinese Medicine (approval number ACU210602).

### Enzymatic digestion of the mesentery

The mesenteric tissue was cut into 1 × 1 mm tissue fragments with sterile surgical scissors and digested in Dulbecco’s modified Eagle’s medium (Invitrogen) containing 1.5 mg·mL^−1^ type II collagenase (C6885; Sigma‐Aldrich, St Louis, MO, USA) and 60 U·mL^−1^ type I DNase (D5025; Sigma‐Aldrich). After 2 h of incubation on a table concentrator (60 r.p.m.) at 37 °C, digestion was stopped with ice‐cold Dulbecco’s modified Eagle’s medium containing 10% fetal bovine serum (Invitrogen) and filtered through a 70‐μm filter. The cell mixture was then centrifuged at 300 ***g*** for 10 min at 4 °C, and the supernatant was removed. Finally, the cell pellet was resuspended in 5 mL of complete medium and seeded.

### Selection of basic medium and optimization of culture conditions

Primary mesenteric cells obtained from at least five mice were cultured in each 10‐cm dish. After stable adhesion of cells, the complete medium was replaced by pericyte conditioned medium (#1201; Science Cell, San Diego, CA, USA), which is a low glucose complete medium supplemented with 2% fetal bovine serum, 1% penicillin–streptomycin and 1% pericyte growth supplement (#1252; Science Cell). Half of the conditioned medium was replaced by an equal volume of fresh medium on alternative days, and floating cell debris was removed.

### Pericyte isolation and purification by magnetic activated cell sorting (MACS)

**Fig. 1:**
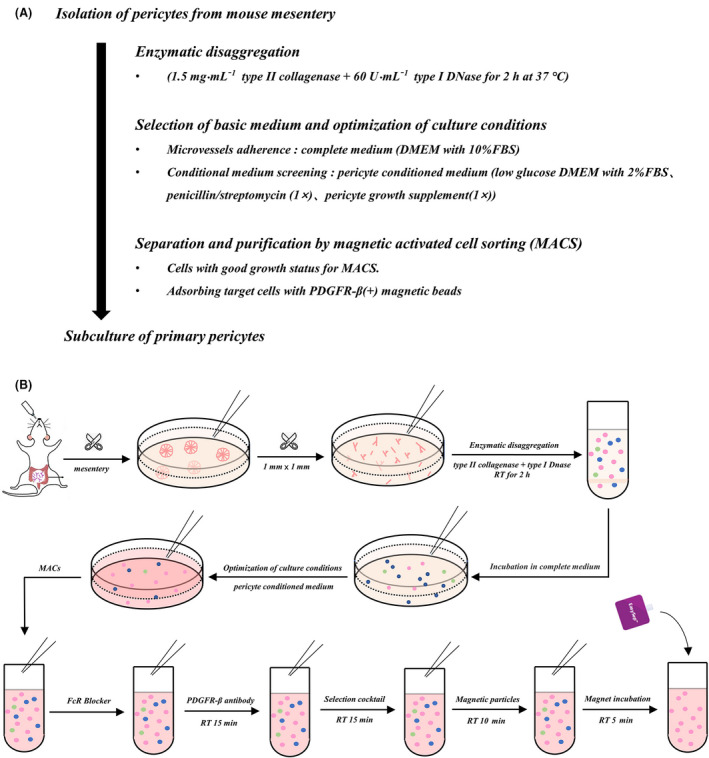
(A) Flowchart of separation of peritoneal vascular pericytes. (B) Schematic protocol for extraction and purification of peritoneal vascular pericytes.

Conditional cultured cells with good growth status were prepared for MACS. A column‐free magnetic sorting platform, called EasySep™ (STEMCELL, Vancouver, BC, Canada), was used to quickly and easily separate cells. After digestion with 2.5 g·L^−1^ trypsin, the number of cells was adjusted to 1 × 10^7^·mL^–1^ and resuspended in 100 μL of the recommended buffer (phosphate‐buffered saline with 2% fetal bovine serum and 1 mm EDTA). One microliter of EasySep™ Mouse FcR Blocker and 3 μL of CD140b/platelet‐derived growth factor receptor‐β (PDGFR‐β) monoclonal antibody (12‐1402‐81; Invitrogen) were added to the cells and mixed. After incubation with the selection cocktail and RapidSpheres™ (a suspension of magnetic particles) (STEMCELL Technologies, Inc., Vancouver, BC, Canada) for 10 min, cells were dissolved and brought to a volume of 2.5 mL. The mixture tube (without a lid) was inserted into the magnet and reacted for 5 min. Finally, the supernatant was discarded and PDGFR‐β^+^ cells were collected from the tube wall. The entire separation protocol is shown in Fig. 1.

### Immunofluorescence staining

A fluorescent double staining kit (G1235; Servicebio, Wuhan, China) based on tyramide signal amplification was performed in accordance with the manufacturer’s instructions. Primary antibodies for histological staining included CD31 (dilution 1 : 5000; GB13428; Servicebio) and PDGFR‐β (dilution 1 : 500; #3169; Cell Signaling, Danvers, MA, USA).

Purified pericytes were fixed, permeabilized, and subsequently blocked with BSA for 1 h. Cells were then incubated with antibodies against α‐SMA (dilution 1 : 100; ab7817; Abcam, Cambridge, UK), PDGFR‐β (dilution 1 : 100; #3169; Cell Signaling), neuron‐glial antigen 2 (NG2) (dilution 1 : 100; sc‐53389; Santa Cruz Biotechnology, Dallas, TX, USA), and CD13 (dilution 1 : 100; sc‐13536; Santa Cruz Biotechnology) overnight at 4 °C. The next day, the cells were washed and incubated with secondary Alexa Fluor 488 anti‐rabbit antibody (dilution 1 : 1000; 4412; Cell Signaling) or Alexa Fluor 594 anti‐mouse antibody (dilution 1 : 1000; 8890; Cell Signaling) for 1 h when protected from light. Nuclear staining was performed with 4′,6‐diamidino‐2‐phenylindole. The images were captured by high‐content analysis (Operetta; PerkinElmer, Waltham, MA, USA). Image analysis and fluorescence quantification were performed using columbus (PerkinElmer) and imagej (NIH, Bethesda, USA).

### Flow cytometry

Adherent cells were digested in 2.5 g·L^−1^ trypsin (25200072; Invitrogen) and dissolved in 15 mL of staining buffer (phosphate‐buffered saline containing 0.2% BSA). After centrifugation at 300 ***g*** for 10 min, the cells were resuspended in 100 μL of staining buffer. Non‐specific binding was blocked with 5% BSA for 1 h, and the cells were then incubated with specific antibodies for 30 min at 4 °C, such as anti‐CD31/PECAM‐1 monoclonal antibody‐fluorescein isothiocyanate (1 µg·test^−1^, 11‐0311‐81; Invitrogen) and anti‐CD140b/PDGFR‐β monoclonal antibody‐PE (1 µg·test^−1^, 12‐1402‐81; Invitrogen). Cells were washed, dissolved, and then detected by flow cytometry (FC500 MPL; Beckman Coulter, Brea, CA, USA). Data acquisition and analysis were performed using an MXP Cytometer and cxp analysis, version 2.1 (Beckman Coulter).

### Cell tubule formation assay

Matrigel matrix (356230; Corning Inc., Corning, NY, USA) was added to a prechilled 96‐well plate at a volume of 50 µL per well. PDGFR‐β^+^ primary mouse peritoneal vascular pericytes (MPVPCs) and CD31^+^ primary mouse peritoneal vascular endothelial cells (MPVECs) purified by MACS were stained with cytomembrane green fluorescent probe (DiO) and red fluorescent probe (DiI), respectively, and then cocultured on Matrigel matrix at a ratio of 1 : 10 per well (pericytes: endothelial cells = 3 × 10^3^ : 3 × 10^4^) for 4 h, after which they formed tube‐like structures. Meanwhile, human retinal microvascular pericytes (HRMPCs) (WN10430; Huaerna Biotech, Wuhan, China) and human retinal microvascular endothelial cells (HRMECs) (HTX2340; Otwo Biotech, Wuhan, China) were analyzed under the same experimental conditions for comparison. CD31^+^ primary MPVEC‐DiI without PDGFR‐β^+^ primary MPVPC‐DiO was cultured at a density of 3 × 10^4^ cells per well on Matrigel matrix for 4 h as a control group, compared to the coculture system. The formation of endothelial tubes was examined by phase‐contrast microscopy and estimated visually by tube length and branch.

### Western blotting

PDGFR‐β^+^ primary MPVPCs were seeded into a six‐well culture plate at a density of 2 × 10^6^·mL^–1^. Transforming growth factor‐β1 (TGF‐β1) (10 ng·mL^−1^) was added and incubated for 24 h. Total proteins were extracted using ice‐cold RIPA buffer containing a phosphatase inhibitor and protease inhibitor cocktail for 15 min. The collected protein was sorted by SDS/PAGE and then electrotransferred to polyvinylidene fluoride membranes. After blocking, membranes were reacted with corresponding primary antibodies at 4 °C overnight and then incubated with horseradish peroxidase‐conjugated anti‐rabbit/mouse immunoglobulin G secondary antibodies for 1 h. Membranes were rinsed and developed with Immobilon Western Chemiluminescent HRP Substrate (WBKLS0500; Millipore, Burlington, MA, USA). Grayscale values were calculated using imagej (NIH) to determine protein expression.

### Statistical analysis

At least three individual experiments were carried out for each object. Data analysis was performed using spss, version 12.0 software (SPSS Inc., Chicago, IL, USA). The significance of the difference between two groups was determined by Student’s *t*‐test. Data are expressed as the mean ± SEM. *P* < 0.05 was considered statistically significant.

## Results

### Anatomical location of mouse mesenteric pericytes

**Fig. 2:**
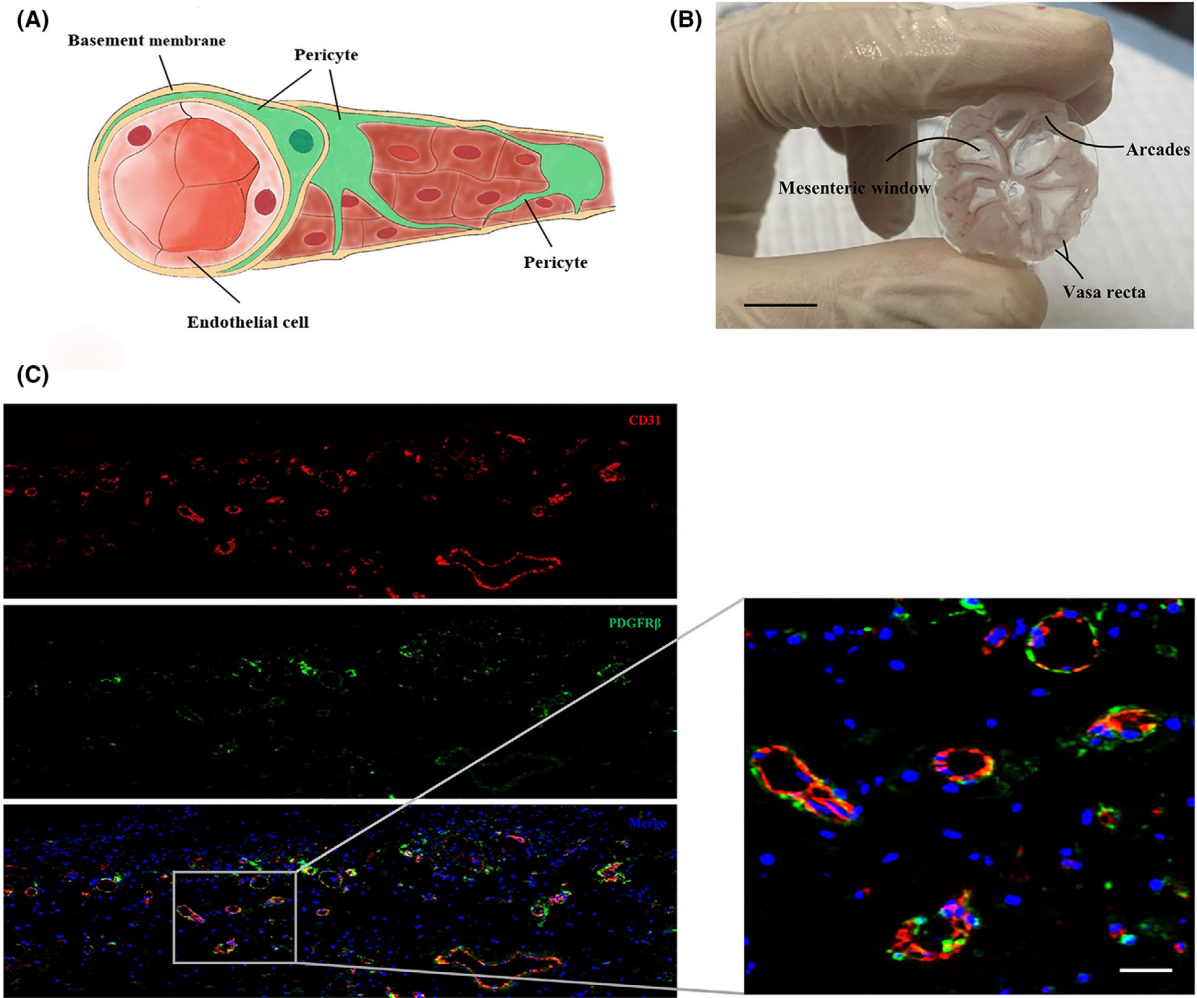
Anatomical location of mouse mesenteric pericytes. (A) Schematic manifestation of pericytes and vascular endothelial cells in microvasculature. (B) Mesenteric tissue extracted from the mouse bowel system (displayed on glass slides). Scale bar = 1 cm. (C) Multiplex immunofluorescence staining with anti‐CD31 and anti‐PDGFR‐β antibodies to validate mouse mesenteric pericytes. Original magnification: 50×. Scale bar = 50 μm.

Pericytes are common in vascular tissue and are embedded in the basement membrane. They surround endothelial cells along the vascular wall and conduct intercellular communication by physical attachment and paracrine signaling (Fig. 2A). The mesentery is a broad, fan‐shaped fold of peritoneum, which mainly consists of loose connective tissue in the middle with mesothelium covering on both sides. Each mesenteric tissue of wild‐type C57BL/6 mice was collected (Fig. 2B) and multiplex immunostained with CD31 and PDGFR‐β, which labeled mesenteric endothelial cells and pericytes, respectively. CD31^+^ vascular endothelial cells formed the vascular internal wall, whereas PDGFR‐β^+^ pericytes encircled vascular endothelial cells. The ratio of endothelial cells to pericytes in mesenteric tissue was approximately 5 : 1 (Fig. 2C). These results suggested that mouse mesentery is a potent and abundant source of primary pericytes.

### Morphological change of primary cells

**Fig. 3:**
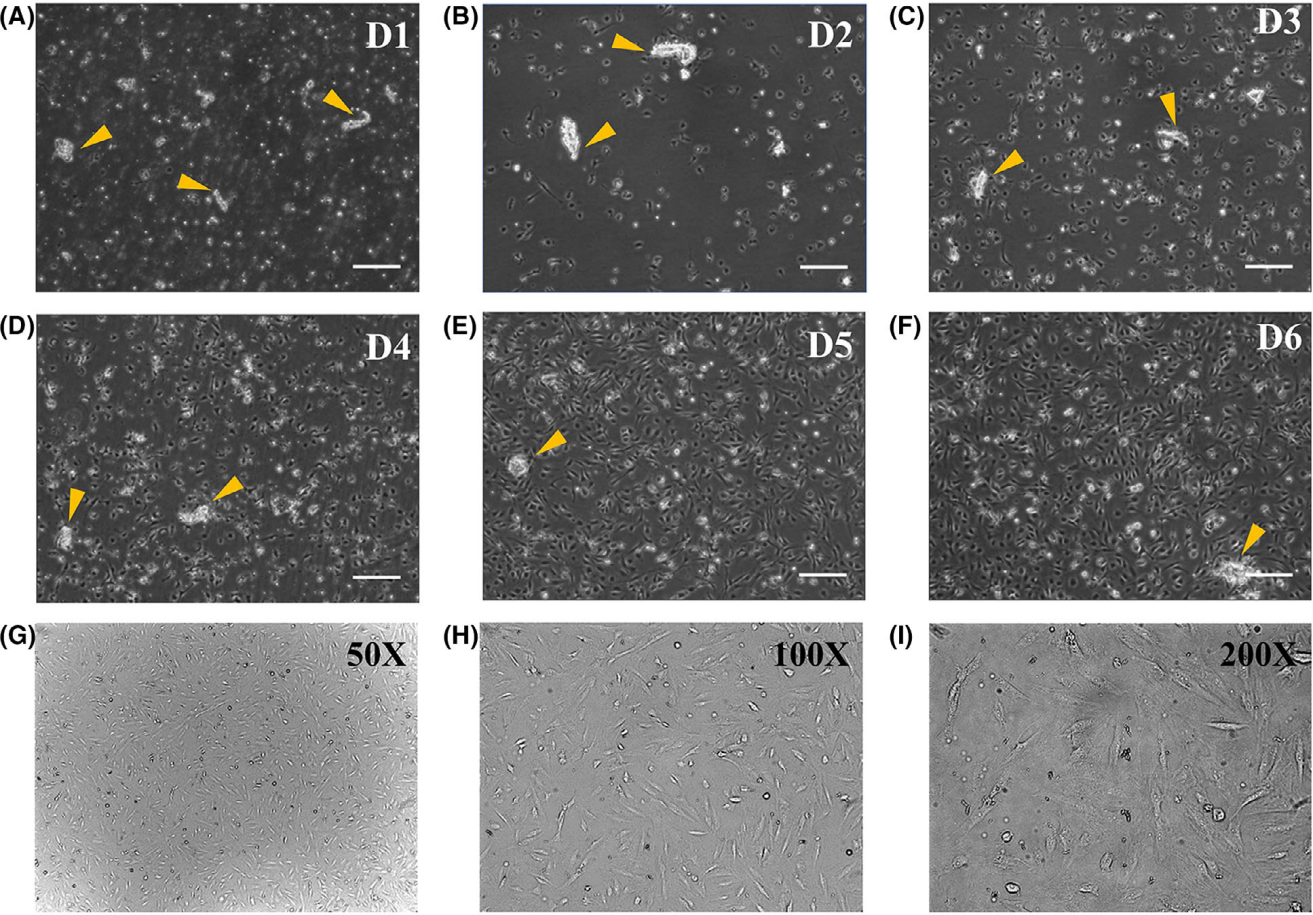
Micrographs of mouse primary peritoneal pericytes under different conditions. (A) Day 1, after enzymatic disaggregation of the mesentery. (B) Day 2, cells became attached, and the medium was changed. (C–F) During days 3–6, cells proliferated and grew to confluence. Scale bars = 250 μm; yellow arrows: microvascular fragments. (G–I) Confluent cell cultures were established after screening with conditioned medium and MACS.

Microvascular fragments were clearly observed in the cell suspension after enzymatic disaggregation, as well as floating endothelial cells, mesothelial cells, and fibroblast‐like cells (Fig. 3A). Seeded cells became adherent to the dish bottom on day 2 (Fig. 3B). Here, pericyte‐conditioned medium was used, half of which was replaced by an equal volume of fresh medium on alternative days. During days 3–6, the adherent cells proliferated and reached 70% confluence. Meanwhile, the microvascular fragment ruptured and surrounding pericytes crawled out (Fig. 3C–F). After consecutive screening by enzymatic digestion and conditional culture, the adherent cells with good status were sorted by MACS. CD31^–^/PDGFR‐β^+^ cells were triangular or polygon with three to five protrusions and grew subconfluently in 3 days. These closely spaced cells exhibited clear contours with elliptical and centered nuclei, and the cytoplasm was homogeneous and transparent (Fig. 3G–I).

### Identification of purified pericytes

**Fig. 4:**
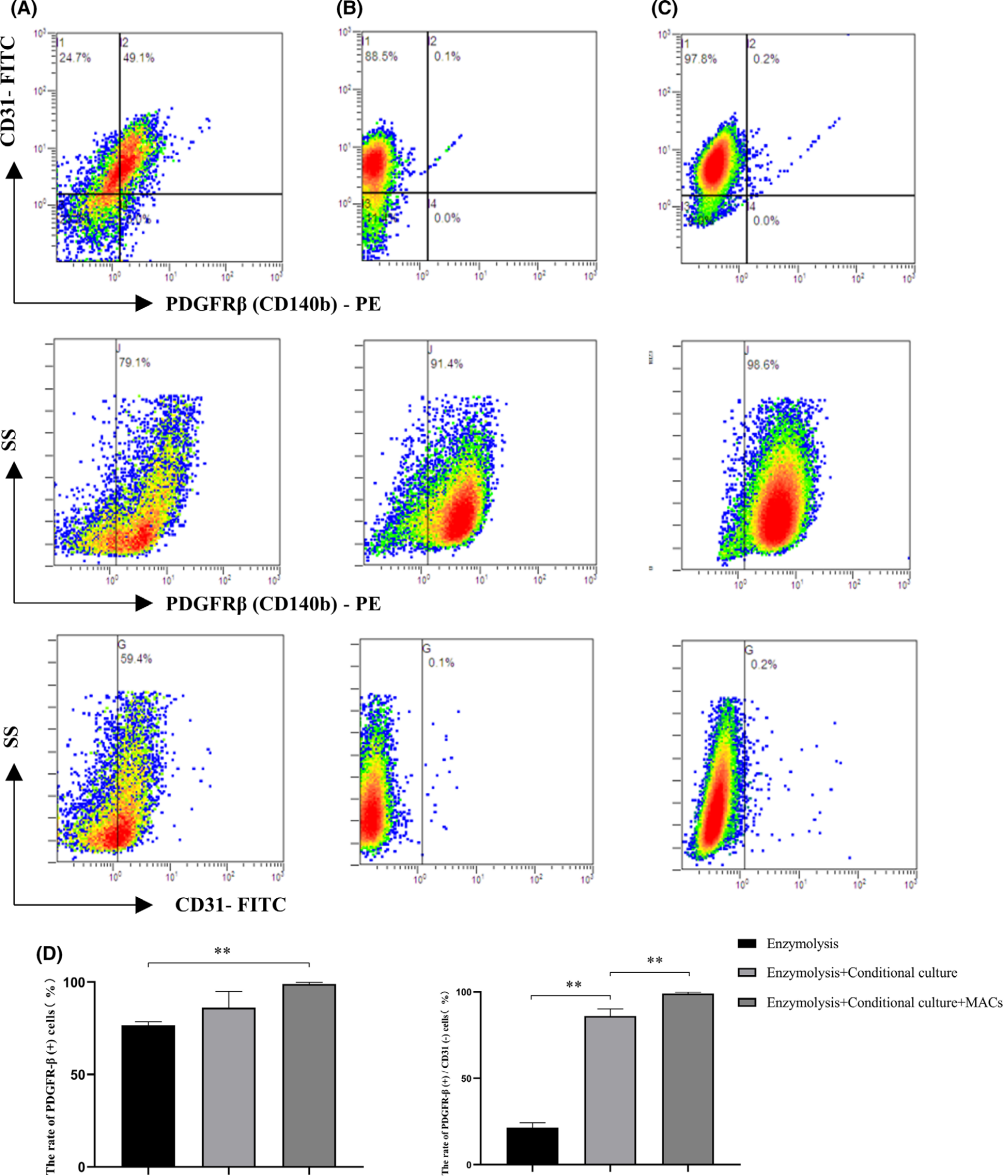
Flow cytometry was used to determine the purity of primary pericytes enriched by different methods. (A) Cells collected by enzymatic digestion alone. (B) Cells screened after pericyte conditional medium. (C) Copurification by conditional culture combined with MACs. (D) The rate of PDGFR‐β^+^ cells (left) and PDGFR‐β^+^/CD31^–^ cells (right) in different protocols (*n* = 3 for each group). Values are expressed as the mean ± SEM. **P < 0.01 (Student’s *t*‐test).

**Fig. 5:**
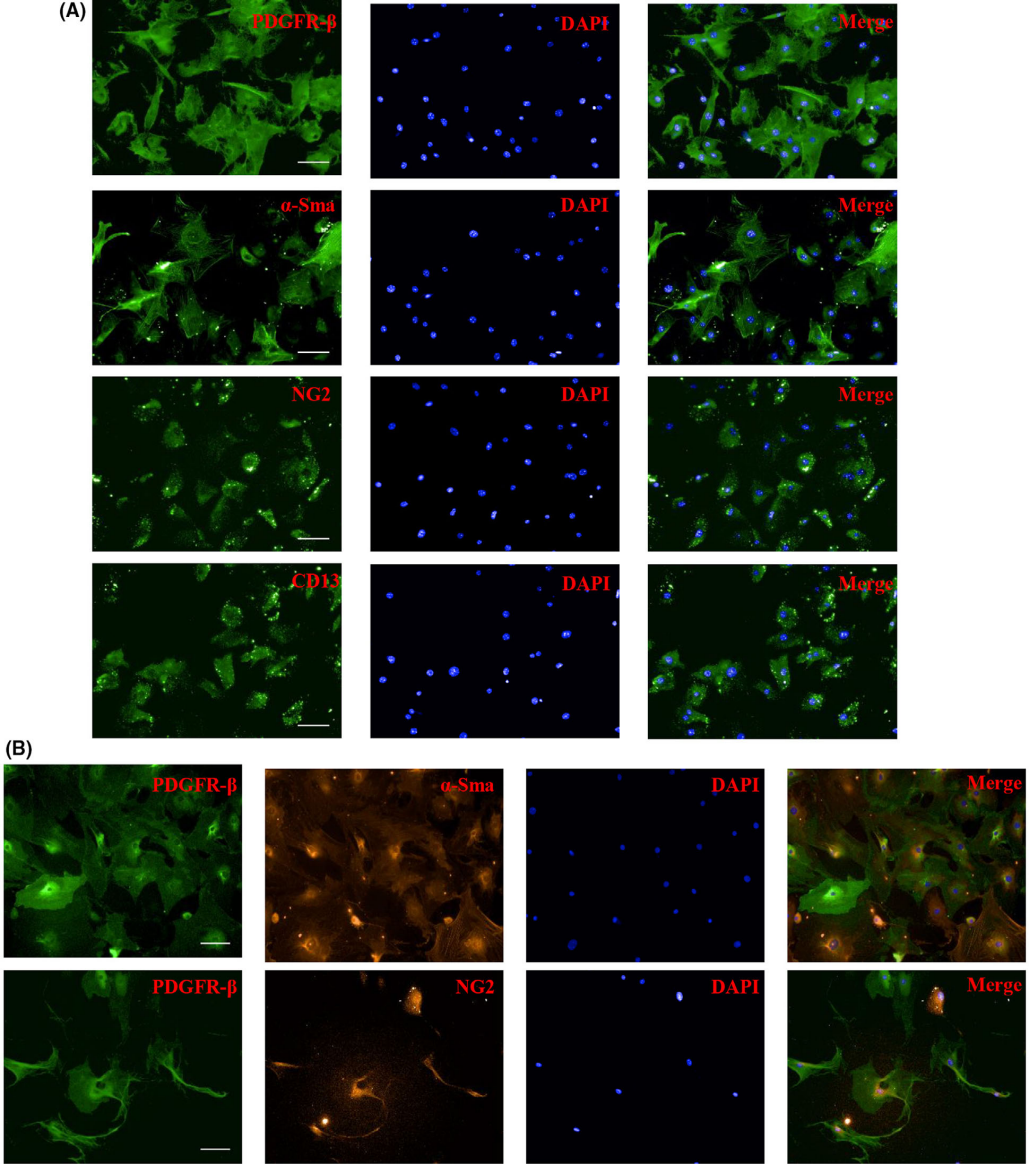
Pericyte markers of the primary cells were stained by immunofluorescence. (A) Primary cells of the third generation were identified by staining for PDGFR‐β, α‐SMA, NG2, and CD13. (B) Double staining for PDGFR‐β/α‐SMA and PDGFR‐β/NG2. Scale bars = 50 μm.

The purity of the enriched primary pericytes was first assessed by flow cytometry. Specific membranous markers of pericytes and endothelial cells were identified as PDGFR‐β^+^/CD31^–^ and CD31^+^/PDGFR‐β^–^, respectively. The results confirmed that the content of CD31^+^ cells was almost 60% by enzymatic digestion alone, whereas PDGFR‐β^+^/CD31^–^ cells represented 21.5 ± 2.8% of the total (Fig. 4A). Combined with pericyte conditional culture, the purity of PDGFR‐β^+^ cells increased (86.0 ± 4.2%) (Fig. 4B). After MACS isolation, the content of PDGFR‐β^+^ cells further increased, and the PDGFR‐β^+^/CD31^–^ cells accounted for 99.0 ± 0.8% of the total (Fig. 4C). The purity of PDGFR^+^ cells extracted in different ways is shown in Fig. 4D. A population of PDGFR‐β^+^/CD31^+^ cells comprised approximately 0.1–0.2% of the total, which may have been a result of non‐specific staining rather than a compensation problem; the proportion was so small, however, that the influence of its error was not considered. We also selected four recognized pericyte markers to identify the primary cells by immunofluorescence staining. The third generation of primary cells was positive for PDGFR‐β, α‐SMA, NG2, and CD13 (Fig. 5A). The cells that positively stained with PDGFR‐β/α‐SMA and PDGFR‐β/NG2 antibodies (Fig. 5B) exhibited confirmed markers of pericytes.

### Coculture of pericytes and endothelial cells promotes tube formation

**Fig. 6:**
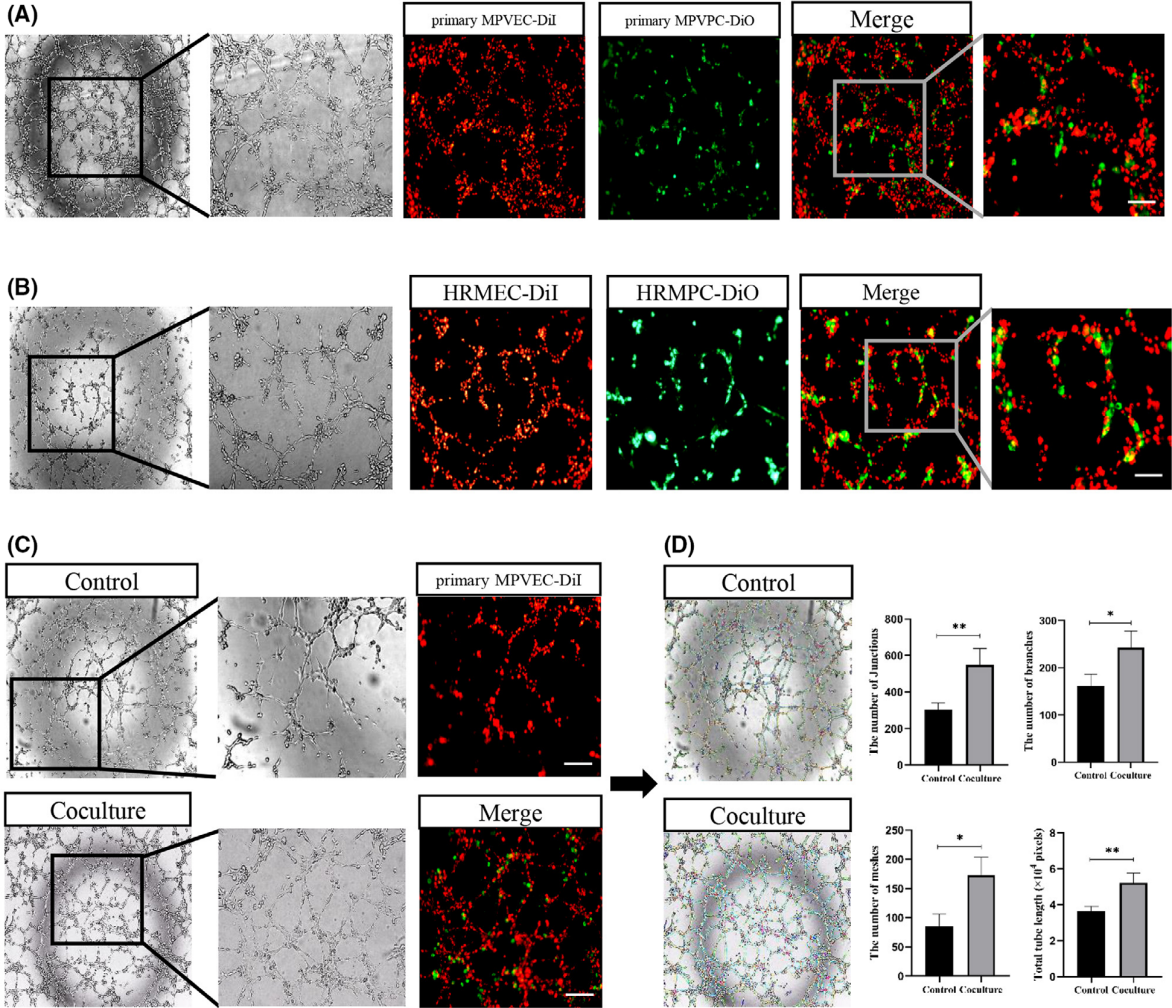
Coculture of pericytes and endothelial cells promotes tube formation. (A) Coculture of primary MPVPC‐DiO with primary MPVEC‐DiI on Matrigel matrix was observed by phase‐contrast microscopy. Scale bars = 300 μm. (B) Coculture of HRMPC‐DiO and HRMEC‐DiI on Matrigel matrix was observed by phase‐contrast microscopy. Scale bars = 300 μm. (C) Coculture of primary MPVPC‐DiO and primary MPVEC‐DiI promoted tube formation compared to simple primary MPVEC‐DiI. Scale bars 600 μm. (D) The coculture system showed higher total tubule length, number of meshes, number of branches, and number of junctions (*n* = 3 for each group). Three fields of view were assessed. imagej was used to obtain the results, and the values are expressed as the mean ± SEM. *P < 0.05, **P < 0.01 versus the control group (Student’s *t*‐test).

An *in vitro* tube formation assay suggested that DiO‐labeled primary MPVPCs were closely bound to DiI‐labeled primary MPVECs in the tubular structure, indicating that pericytes share a basement membrane with endothelial cells (Fig. 6A). HRMPC‐DiO and HRMEC‐DiI were compared and demonstrated similar results (Fig. 6B). Under the same cell density and incubation time conditions, coculture of PDGFR‐β^+^ primary MPVPC‐DiO and CD31^+^ primary MPVEC‐DiI could promote tube formation compared to simple CD31^+^ primary MPVEC‐DiI, which was significantly increased in total tubule length, number of meshes, number of branches, and number of junctions (Fig. 6C,D).

### TGF‐β1 promotes the transition of pericytes into myofibroblasts

**Fig. 7:**
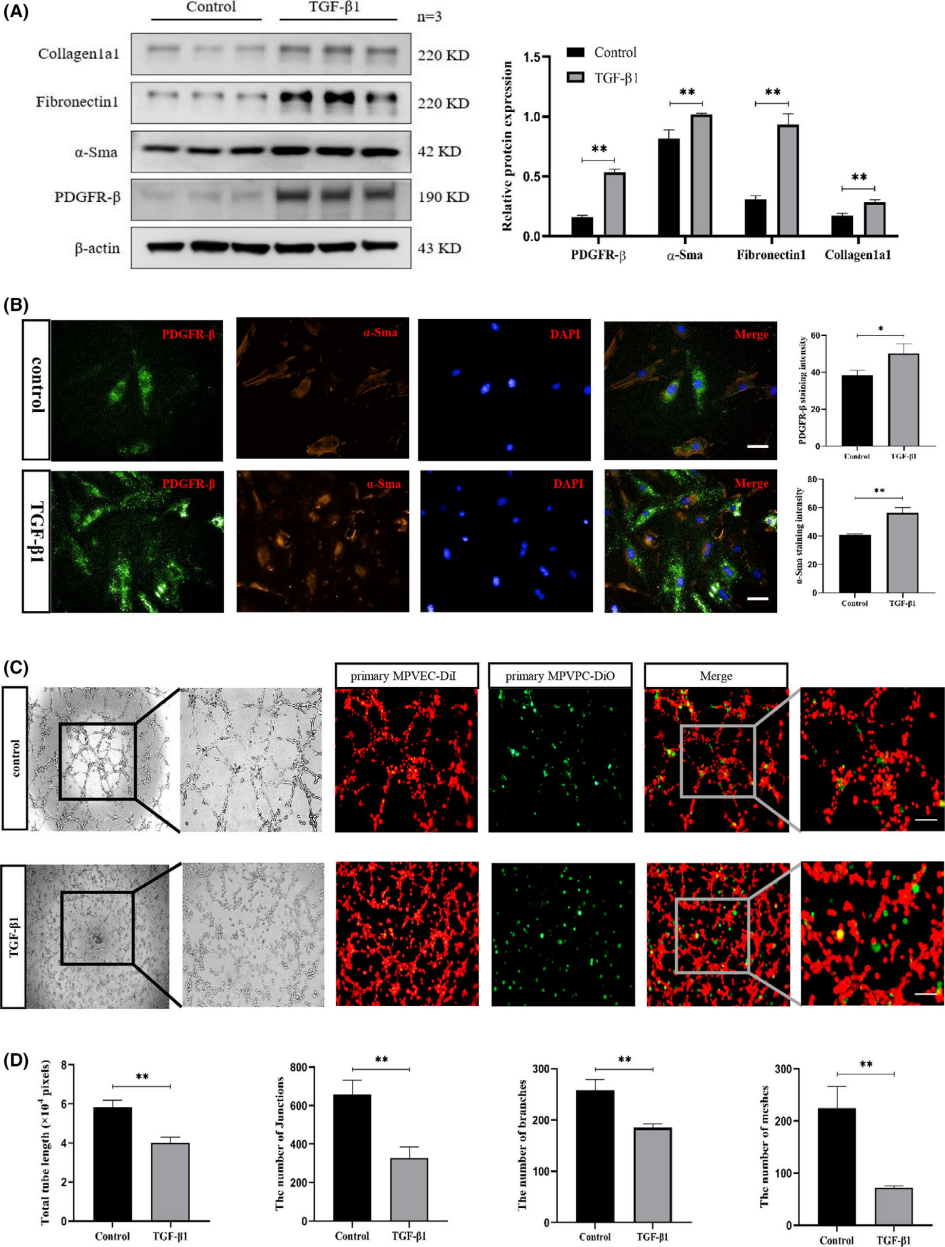
TGF‐β1 induces the transition of pericytes to myofibroblasts. (A) Western blotting was used to detect the protein expression of PDGFR‐β, α‐SMA, fibronectin 1 and collagen 1a1. (B) Immunofluorescence staining displayed changes in the fluorescence intensity of PDGFR‐β and α‐SMA. Scale bars = 50 μm. (C) Coculture of primary MPVPC‐DiO with primary MPVEC‐DiI on Matrigel matrix was observed by phase‐contrast microscopy. Scale bars = 300 μm. (D) Cell tubule formation was examined by total tube length, number of junctions, number of branches, and number of meshes (*n* = 3 for each group). Three fields of view were assessed. imagej was used to obtain the results, and the values are expressed as the mean ± SEM. *P < 0.05, **P < 0.01 versus the control group (Student’s *t*‐test).

We examined phenotypic changes in primary pericytes after TGF‐β1 stimulation. The protein expression levels of PDGFR‐β, α‐SMA and fibrosis markers, such as fibronectin 1 and collagen 1a1, were significantly elevated after TGF‐β1 treatment (10 ng·mL^−1^ for 24 h) (Fig. 7A). Immunofluorescence staining also demonstrated TGF‐β1‐induced enhancement of PDGFR‐β and α‐SMA (Fig. 7B). Meanwhile, we observed an effect of TGF‐β1 on pericyte‐regulated tube formation. The rate of epithelial tubular formation was significantly reduced after TGF‐β1 stimulation, and DiO‐labeled pericytes were clearly separated from tube‐like structures formed by endothelial cells (Fig. 7C,D).

## Discussion

For the last few years, the combination of multilabel microscopy and genetic state‐of‐art technology has enabled striking progress in the exploration of novel functions for pericytes in health and disease. In the field of fibrosis, emerging studies have suggested that pericytes are progenitors of myofibroblasts/fibroblasts and contribute to the deposition of ECM [12, 13]. Scientists have found that MyoFs do not mainly originate from tubular epithelial cells in a unilateral ureteral obstruction model. Instead, most are derived from renal interstitial perivascular cells, and PDGFR‐β^+^/α‐SMA^–^ pericytes gradually transform into α‐SMA^+^ MyoFs [14]. During homeostasis and fibrosis of human and mouse kidneys, pericyte and fibroblast subsets were identified as the main sources of MyoFs, whereas injured renal tubular epithelial cells, endothelial cells, and monocytes exhibited only minor ECM expression [8]. Admittedly, pericytes may be a highlighted source of MyoF in the peritoneal fibrosis process. Blocking the pathological process of pericyte–myofibroblast transition would provide a new approach for alleviating peritoneal fibrosis.

At present, the research on pericytes mainly relies on animal models. Although we observed a dynamic process of generation, proliferation, migration, and transition in pericytes, animal models are always time‐consuming and require expensive experiments. In addition, cell signal transduction research is easier to perform in cell lines. To our knowledge, no peritoneal pericyte cell line or protocol for cells has been established. Therefore, an innovative extraction method is proposed to effectively collect primary peritoneal pericytes, which maintain their biological characteristics *in vivo*.

The ratio of pericytes to endothelial cells in vascular system varies from 1 : 1 to 1 : 10 according to the respective tissue and animal species [15, 16], as well as the characterization technique. Pericytes account for a very low portion of any sample. Elimination of the nonvascular structure before separation optimizes purity and cell yield. Thus, we selected mesentery with abundant vascularity that supplies the intestine as a potential sample, as opposed to diaphragmatic or parietal peritoneum that contain a large amount of muscle fibers. A large‐area capillary network provided sufficient ultrafiltration in dialysis and sufficient pericytes for identification. The enzymatic digestion was used first. By contrast to protocols for other samples, trypsin digestion [17] was replaced by moderated digestion of type II collagenase combined with type I DNase. Collagenase exhibited a potent dissociation effect on interstitial/stromal cells, detaching them from collagen without damage. Moreover, the additional use of type I DNase could effectively prevent cell agglutination induced by degraded DNA in the separation process [18]. By centrifugation and filtration with 70‐μm cell strainers, we successfully dissociated the microvascular fragments and removed undigested tissue fragments and masses of adipocytes.

Most studies have aimed to isolate pericytes from retinal or brain microvasculature. Vascular cells obtained from adult mouse brains were initially cultured in regular medium of brain endothelial cells. After two generations, the cells were transferred to a medium suitable for the growth of pericytes. Cells were gradually replaced by pericytes with high purity [19]. As a result of the complex composition of the mesentery, endothelial and peritoneal mesothelial cells were the main shedding cells in mesenteric microvasculature except for pericytes, which was different from astrocyte contamination in brain microvasculature [20]. Therefore, suitable conditioned medium was required. Low concentrations of glucose and fetal bovine serum could effectively inhibit the growth of endothelial and mesothelial cells. Supplementation with pericyte growth factor clearly promoted pericyte proliferation [20]. The growth of pericytes also inhibited the proliferation of other ingredients when the culture conditions changed, which was confirmed in the present study by flow cytometry. Fluorescence‐activated cell sorting, differential centrifugation and MACS are common methods for cell separation [21]. Fluorescence‐activated cell sorting is time‐consuming and easily contaminated, and the survival rate of cultured cells is low. Differential centrifugation may cause great mechanical damage to primary cells and affect cell activity. Therefore, MACS based on the antigen‐antibody binding reaction was selected in the present study, which enabled high purity vascular pericytes, and also minimized the impact on cell viability because of its simple operation.

It is imperative to characterize populations of pericytes as a result of their functional and phenotypical heterogeneity. Because the cells are pluripotent, they require monitoring for pericyte differentiation [22]. Pericytes express a series of cell proteins, but lack specific markers. These markers adjust their expression levels with respect to growth and development, pathological reactions, and culture conditions of pericytes. The membrane‐bound markers include PDGFR‐β, CD13, NG2, CD146, and endoglin. Cytosolic markers for pericyte characterization include α‐SMA, nonmuscle myosin, desmin, nestin, and vimentin. Specific inhibition of the PDGFR‐β signaling pathway could eliminate perivascular mature pericytes in tumors, leading to vascular hyperdilation. This phenomenon has also been observed in the vascellum of the central nervous system, with an almost completely absence of pericytes during vascular development in PDGF‐B knockout mice [23]. In addition, in rodent animals, α‐SMA is expressed only in mature pericytes rather than in early developmental stages [24]. Thus, the maturation degree of different types of pericytes can be classified by measuring the percentage of NG2^+^ or PDGFR‐β^+^ cells expressing α‐SMA [25]. NG2, a transmembrane protein that interacts with the cytoskeleton and enhances signal transduction through integrins and receptor tyrosine kinase growth factor receptors, affects the proliferation and motility of pericytes. At least 97% of pericytes express PDGFR‐β, whereas NG2^+^ pericytes account for 50–80% [24]. Pericyte‐specific NG2 ablation resulted in vascular structural deficits, including reduced pericyte ensheathment of the endothelium and repressed formation of the pericyte/endothelial network [26]. Thus, the emerged PDGFR‐β and NG2 can serve as convenient and reliable pericyte markers to confirm the existence of these mural cells [24]. In the present study, the pericyte surface antigen PDGFR‐β was used to target cells by magnetic beads. The isolated mature pericytes (day 3) displayed classical morphology and were positive for NG2, α‐SMA, PDGFR‐β, and CD13, verifying the high efficacy, time savings, and low cost of our protocol.

Pericytes share a basement membrane with endothelial cells that regulates the development, remodeling, and permeability of the vascular system through a variety of intercellular junctions, whereas their finger‐like protrusions regulate blood flow to capillaries. We simulated angiogenesis using a Matrigel matrix tube formation assay to observe the morphological characteristics of pericytes in tube‐like structures. The results showed that coculture of primary pericytes and endothelial cells could promote tube formation compared to simple primary endothelial cells. In addition, a fluorescence probe was used to trace the migration of pericytes during tubule formation, and it was found that pericytes were closely combined with endothelial cells in the tubular structure, consistent with the physiological structural characteristics of pericytes and endothelial cells. Pericyte loss can lead to structural damage and impaired regeneration of microvasculature, although there are very few studies on the mechanism of pericyte loss in peritoneal dialysis environments. Studies have suggested that excessive accumulation of advanced glycation end products or glucose degradation products during peritoneal dialysis interferes with pericyte recruitment to endothelial cells during new vascular formation, resulting in high peritoneal permeability in patients [27]. High glucose induces macrophages to secrete interleukin‐1β, which promotes the transition of pericytes into fibroblasts. This result was confirmed in glucose peritoneal dialysis mice and liopolysaccharide‐induced NG2^+^ pericytes [28]. We were therefore reasonably confident that peritoneal microvascular pericyte loss and vascular structural and functional destruction exist in the peritoneal dialysis environment with high glucose, high osmolarity, advanced glycation end products, and inflammatory factors. Pericyte‐endothelial cell crosstalk results from the exchange of cytokines between two cell types during angiogenesis. PDGF‐BB, PDGF‐DD, endothelin‐1, and TGF‐β1 produced by endothelial cells are the main driving factors of pericyte activation, which trigger pericyte movement through interactions with multiple pericyte surface PDGFRs, endothelin receptors, and TGF‐βRs [29]. Meanwhile, PDGF‐BB induces phosphorylation of downstream PDGFR‐β to promote TGF‐β1 transcription in pericytes [30], which is responsible for inducing endothelial cell quiescence as a component of vascular maturation. Thus, TGF‐β1 plays an important role in pericyte‐endothelial cell crosstalk. TGF‐β1 was found to induce pericyte injury and stimulate the transformation of pericytes into a myofibroblast phenotype. We pretreated primary pericytes with TGF‐β1 and found that the expression of PDGFR‐β and α‐SMA was significantly increased, suggesting that pericytes were activated to some extent and transformed into a myofibroblast phenotype. On this basis, the cells were cocultured with endothelial cells, and the results showed that tubule formation was reduced, suggesting that endothelial cells reduced pericyte recruitment after TGF‐β1 stimulation. Meanwhile, the primary pericytes were clearly separated from the endothelial tubules, and the tube‐like structures were destroyed. All of the above results suggested that extracted primary pericytes have the potential to promote endothelial cell tubulogenesis and transition and could be potent cell carriers for pericyte–myofibroblast transition studies.

In conclusion, our protocol provides a feasible method for obtaining high purity primary peritoneal pericytes in mice, which have good cell activity and stable biological functions.

## Conflict of interests

The authors declare that they have no conflicts of interest.

## Author contributions

MS conceived and designed the experiments. LT and JS performed the experiments and wrote the manuscript. MY revised the manuscript. YS contributed reagents/materials/analysis tools. JZ analyzed the data.

## Data Availability

All data generated or analyzed during this study are available within this article. Further inquiries can be directed to the corresponding author.
